# How Much Data Is Enough? A Reliable Methodology to Examine Long-Term Wearable Data Acquisition in Gait and Postural Sway

**DOI:** 10.3390/s22186982

**Published:** 2022-09-15

**Authors:** Brett M. Meyer, Paolo Depetrillo, Jaime Franco, Nicole Donahue, Samantha R. Fox, Aisling O’Leary, Bryn C. Loftness, Reed D. Gurchiek, Maura Buckley, Andrew J. Solomon, Sau Kuen Ng, Nick Cheney, Melissa Ceruolo, Ryan S. McGinnis

**Affiliations:** 1 M-Sense Research Group, University of Vermont, Burlington, VT 05405, USA; 2 Medidata Solutions, A Dassault Systèmes Company, New York, NY 10014, USA; 3 Department of Bioengineering, Stanford University, Stanford, CA 94305, USA; 4 Department of Neurological Sciences, University of Vermont, Burlington, VT 05405, USA

**Keywords:** wearable sensors, remote monitoring, gait, postural sway, neurological disorders

## Abstract

Wearable sensors facilitate the evaluation of gait and balance impairment in the free-living environment, often with observation periods spanning weeks, months, and even years. Data supporting the minimal duration of sensor wear, which is necessary to capture representative variability in impairment measures, are needed to balance patient burden, data quality, and study cost. Prior investigations have examined the duration required for resolving a variety of movement variables (e.g., gait speed, sit-to-stand tests), but these studies use differing methodologies and have only examined a small subset of potential measures of gait and balance impairment. Notably, postural sway measures have not yet been considered in these analyses. Here, we propose a three-level framework for examining this problem. Difference testing and intra-class correlations (ICC) are used to examine the agreement in features computed from potential wear durations (levels one and two). The association between features and established patient reported outcomes at each wear duration is also considered (level three) for determining the necessary wear duration. Utilizing wearable accelerometer data continuously collected from 22 persons with multiple sclerosis (PwMS) for 6 weeks, this framework suggests that 2 to 3 days of monitoring may be sufficient to capture most of the variability in gait and sway; however, longer periods (e.g., 3 to 6 days) may be needed to establish strong correlations to patient-reported clinical measures. Regression analysis indicates that the required wear duration depends on both the observation frequency and variability of the measure being considered. This approach provides a framework for evaluating wear duration as one aspect of the comprehensive assessment, which is necessary to ensure that wearable sensor-based methods for capturing gait and balance impairment in the free-living environment are fit for purpose.

## 1. Introduction

Wearable sensors are increasingly common, with a vast number of uses including health research [1,2,3,4,5,6,7,8,9,10,11] and fitness tracking [12,13,14]. Laboratory-based studies of features of gait and postural sway have contributed important foundational knowledge to the field of wearable sensor-based movement tracking [2,15,16,17,18,19,20]. However, they also indicate that movement characteristics measured in the lab often do not reflect those displayed during daily life, and thus only capture a limited picture of balance and mobility impairment [4,21,22]. Recent advances in wearable technology pair improvements in battery life with conformal designs [23], allowing studies to be deployed in the free-living environment. Work in this emerging area has focused largely on demonstrating feasibility [24] and identifying reliable measures of gait performance [1,21].

Many free-living studies of balance and mobility have been conducted in older adults; however, these studies may be more informative in certain clinical populations, such as those with neurological disorders [9,25]. For example, persons with multiple sclerosis (PwMS) experience symptom fluctuations due to disease. As a result, a bi-annual clinic visit or in-lab assessment may not capture an accurate picture of their impairment, nor its variability [26]. In contrast, remote observation with wearable sensors could enable a more accurate assessment of balance and mobility impairment that is sensitive to variability over time and is captured while patients are engaging in their everyday lives. However, it is not yet clear how long we must monitor these patients to capture an accurate picture of their impairment and its variability.

Prior studies have found that the necessary wear duration for capturing measures of mobility impairment depends on the activity, metric, and population being considered. For example, 2 to 3 days of data are required to capture gait speed, and 4 days are required to capture daily step counts in healthy adults [22,27]. Three days are needed for remotely monitoring chair stand tests in healthy adults and persons with Parkinson’s disease [28]. Between 2 and 7 days are required for physical activity metrics (e.g., actigraphy) depending on which metric is being considered [29,30,31,32,33,34]. While these studies recommend sensor wear durations, there is no established method for arriving at these conclusions. Some studies rely on intra-class correlations (ICC) for this analysis [22,28]. Others use analysis of variance [31], generalizability theory [33] (similar to ICC) or a combination of ICC and difference testing [32]. Each of these approaches considers different aspects of the data, leading to slightly different conclusions. Moreover, these methods do not consider how wear duration impacts the relationship between sensor-derived parameters and other important variables, such as patient-reported measures of impairment. This burgeoning field of research has only considered a small subset of potential wearable-derived metrics for characterizing balance and mobility impairment, and a standardized and rigorous process for evaluating necessary wear duration remains an unmet need.

For PwMS, prior work has identified key laboratory-based measures of balance and mobility impairment that can be derived from wearable sensor data. Spatiotemporal gait metrics, such as gait speed and stride time, have been associated with disease severity [21,35] and fall risk [2]. Similarly, postural sway metrics have been associated with fall risk and balance impairment in PwMS [3,19]. Given these findings, it is likely that remote monitoring of gait and postural sway could be important in this population. However, to the best of our knowledge, it remains unclear how long PwMS must be monitored in the free-living environment to provide reliable measurements of these parameters.

The purpose of this work is to demonstrate a comprehensive and reproducible approach for determining the wearable monitoring duration required to capture an accurate picture of impairment and its variability. We aim to establish this minimum monitoring period to balance patient burden, convenience, and cost. We apply this approach to study the impact of wear duration on postural sway and gait measures in PwMS.

## 2. Materials and Methods

**Figure 1:**
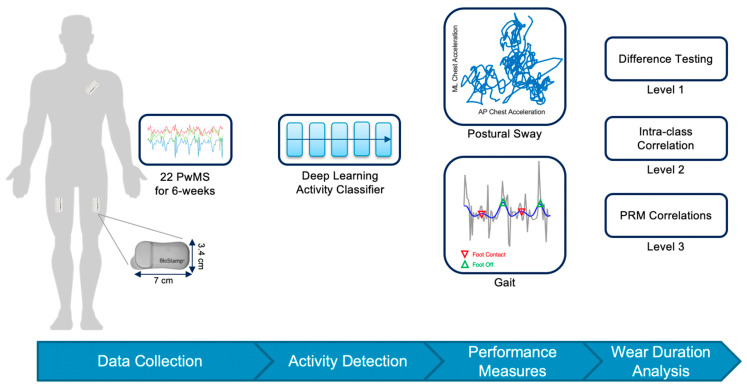
Overview of approach for wear duration analysis. Accelerometer data were collected from BioStamp nPoint^®^ (Medidata) devices worn on the chest and bilaterally on the thighs by a sample of persons with multiple sclerosis (PwMS) for 6 weeks. Sensors were worn for all hours of the day during monitoring periods. A deep learning approach was used to detect bouts of walking and standing from which performance measures were extracted. Measures were analyzed through a three-stage process to determine the number of days of wear required to capture an accurate picture of impairment and its variability.

An overview of the approach used for studying the wear duration required for capturing gait and balance impairment and its variability with wearables in a sample of PwMS is provided in Figure 1. As depicted, we remotely collected data from PwMS, used a classification model to identify periods of walking and standing, computed metrics of gait and sway, and then performed our analysis of wear duration. The sample and associated experimental protocol are detailed in Section 2.1. The framework for detecting walking and standing bouts and extracting associated balance and mobility performance parameters is presented in Section 2.2. Finally, the three-stage wear duration analysis is presented in Section 2.3 along with details for how it was implemented in this study.

### 2.1. Participants and Protocol

Herein, we consider data from 22 PwMS (5:16 Male:Female, mean ± standard deviation, age 51 ± 9 y/o) recruited from the Multiple Sclerosis Center at University of Vermont Medical Center and from the University of Vermont’s IDEAL for MS Program (inclusion: No condition affecting balance and mobility other than MS, ambulatory without aid, no known skin hypersensitivity to adhesives or hydrogel, not pregnant or breastfeeding).

Participants were asked to complete biweekly at-home sensor wear for 12 weeks, yielding 6 weeks of sensor data for analysis. All participants completed at least 2 weeks of monitoring (n = 22), 21 participants completed 5 weeks, and 19 completed all 6 weeks of monitoring. During the sensor wear weeks, participants were asked to complete a daily 30-s chair stand test, a 1-min walk, and a 30-s standing balance assessment (upright and still for 30 s). Each evening participants were asked to complete a daily falls survey and the Activity-Specific Balance Confidence assessment (ABC) [36]. At the end of each non-sensor wear week, participants were asked to complete the Modified Fatigue Impact Scale (MFIS) [37] and 12-item Multiple Sclerosis Walking Scale (MSWS) [38]. This timing was chosen since these surveys ask participants to recall the past 2 weeks. During active sensor wear weeks, participants were instrumented with BioStamp nPoint sensors for all hours of the day located on the left upper chest, and bilaterally on the anterior aspect of each thigh collecting acceleration (31.25 Hz, ±16 G) and electromyography (250 Hz) data. This sensor system is FDA cleared and details of these sensors have been previously discussed [39]. Data from the sensors were saved locally on the sensors and then uploaded to the nPoint cloud via a provided dock following a daily sensor change. Moreover, participants were asked to fill out the Patient-Determined Disease Steps (PDDS) following the completion of their monitoring period [40]. Due to the remote nature of the study, patient disability was assessed with PDDS rather than a neurologist-conduct Expanded Disability Severity Assessment [41]. The mean (std) survey results for our cohort are as follows: PDDS 0.88 (1.05); ABC 77.6 (21.9); MFIS 28.3 (16.1); MSWS 19.1 (7.1). This protocol was approved by the University of Vermont’s Institutional Review Board (CHRMS 21-0401).

### 2.2. Remote Analysis Pipeline

Periods of walking and standing were identified in the wearable accelerometer data from each participant using a classification model as described previously [4]. Briefly, this model uses a deep learning approach where windows of raw accelerometer data from the thigh and chest are classified as walking, standing, sitting, lying or other using a model with two Long-Short-Term-Memory layers [4,42]. This model was trained on over 100,000 4-s observations of acceleration from a different cohort of PwMS, healthy adults, and persons with Parkinson’s disease resulting in a validation accuracy over 96%.

Following activity classification, gait events were identified using the thigh acceleration-based method as described in previous work [1,43]. Walking bouts with two or more valid strides were used for the analysis. Temporal, stability, and asymmetry measures of gait were extracted from each bout. The temporal gait parameters (computed for each stride and averaged across the bout) considered were stride duration, stance duration, swing duration, duty factor, and double support duration [1]. The gait asymmetry parameters considered were duty factor asymmetry (normalized using the L1-norm, $\mathrm{Asymmetry}=\frac{\left|\mathrm{Right}-\mathrm{Left}\right|}{0.5\left(\left|\mathrm{Right}\right|+\left|\mathrm{Left}\right|\right)}$), an affine transformation of the correlation between the right thigh and left thigh raw acceleration in order that a result of one corresponds to a correlation of zero with the transformation (Correlation Asymmetry=0.5∗(1−corr(right,left)), and the asymmetry of an ensemble average of stride acceleration between the right and left leg normalized by the L1-norm method (Acceleration Asymmetry) [1]. The gait stability parameters considered were the root mean square (RMS) of the anterior-posterior (AP) acceleration (RMS AP), frequency dispersion of the media-lateral (ML) acceleration [18], entropy ratio between the trunk and thigh [44], and Lyapunov Exponent of the AP and ML directions [18]. The entropy ratio was only calculated for walking bouts longer than 30 s, and the Lyapunov Exponent was only calculated for walking bouts longer than 1 min. Entropy ratio asymmetry normalized using the L1 method was also calculated.

Measures of postural sway were extracted from all standing periods of 30-s or longer and are described in detail in [3,45]. For consistency with the development of the postural sway measures and algorithm, standing periods of at least 30-s were used. Briefly, the accelerometer magnitude in the horizonal plane during each standing bout was used to inform the postural sway parameters sway area (normalized by bout duration), centroidal frequency, distance, 50th percentile of the frequency content, 95th percentile of the frequency content, frequency dispersion, jerk, mean period, mean velocity, path (normalized by bout duration), power in the frequency spectrum, range of acceleration, and rms of acceleration. Sway distance thresholds were used to identify and remove standing periods that were completely still (e.g., participant was leaning on something) or extremely erratic. Thresholds were set using the 98.5th and 1.5th percentile of random ten-thousand standing bouts from the sample. Several thresholds were tested and visually inspected to ensure that valid standing periods were not removed. Both gait and sway features were computed from custom MATLAB scripts using Medidata’s Sensor Cloud Network Analytics service, a cloud-based computing platform designed to run third-party algorithm code against raw sensor data at scale.

### 2.3. Wear-Time Analysis

We propose a three-stage process for analyzing the wear duration required for capturing impairment and its variability with wearables. The stages include (1) difference testing, (2) intra-class correlation (ICC), and (3) correlation to established clinical measures. These three stages fit nicely into the emerging framework for identifying digital medicine technologies that are fit for purpose [25,46]. Specifically, Stages 1 and 2 are key aspects of Analytical Validation, which aims to establish the performance of algorithms that translate raw sensor data into measures of human physiology or behavior. Stage 3 is a key aspect of Clinical Validation, which aims to demonstrate that a digital medicine technology captures the phenotype of interest in the intended clinical population. To inform the proposed three-stage analysis, the researcher must first partition their dataset into candidate wear durations (e.g., 1 h, 1 day, 1 week, 1 month) and choose a duration (baseline) they aim to compare against. The baseline should be wear duration, which is expected a priori to capture impairment and its variability for the measures being considered. In Stage 1, statistical difference testing (e.g., rank sum tests) is used to identify the wear durations that demonstrate significant differences in the median, 95th percentile or variability (coefficient of variation—CV) relative to baseline. In Stage 2, intra-class correlation analysis is used to identify the reliability of measures extracted from each wear duration relative to baseline. We recommend conducting this analysis with the median, 95th percentile, and variability of each measure, to capture the central tendency and edges of the distribution, using a ‘C-k’ or similarity type correlation, as performed previously, with the addition of CV to capture variability [22]. Wear durations that demonstrate an ICC of 0.7 and higher provided reliable measures. In Stage 3, the correlation (e.g., Spearman rank correlation) between measures extracted from each wear duration and established clinical measures are examined. Wear durations that yield significant correlations with established clinical measures are said to capture the clinical phenotype of interest in the intended patient population.

For the dataset of PwMS we considered here, 1 week of data served as the baseline, and we examined wear durations of 1, 2, or 3 days, and 2, 3, 4, 5, and 6 weeks in Stages 1 and 2. For Stage 3, we examined wear durations between 1 and 14 days. For the statistical analysis, we leveraged non-parametric rank sum testing with a significance threshold of 0.05 for Stage 1 and ‘C-k’ type ICCs (threshold of 0.7 indicating a strong ICC) for Stage 2. For Stage 3, we computed Spearman rank correlations between sensor-derived measures (median, 95th percentile, and CV) at each wear duration and the ABC (median over 2 weeks) and MFIS (sampled at the end of 2 weeks). The ABC captured balance confidence and has been shown to relate to fall risk and other functional-assessments [47,48]. The MFIS captured fatigue [49], which has been shown to relate to fall risk [48], and is widely used clinically [50]. We considered the emergence of a significant correlation followed by similar strength correlations to be a reliable estimate for required wear duration. A power analysis was performed on selected features to determine the stability of these findings. This was carried out using a bootstrap with 1000 replicates and comparing the synthetic data between two durations [51].

The outlined three-stage wear duration analysis allows us to identify the number of days of wear required for capturing impairment and its variability with wearables. However, it is important that we also understand what factors may impact wear duration in order that we can better predict how many days may be required. To this end, we employed regression analysis to investigate our hypothesis that longer wear periods are required for measures with relatively few observations or with high variability during a given day. We operationalized this hypothesis by defining the wear duration as the number of days required to yield no differences relative to baseline and strong ICCs for a given physiological measure. The number of observations was captured by considering the average number of times a measure was computed per day and variability was captured with the CV of the measure per subject across a 2-day period.

## 3. Results

Examining the outcome of the three-stage wear duration analysis, we first report the results of the difference testing (Diff) and intra-class correlation (ICC) analysis for measures of gait (Section 3.1) and postural sway (Section 3.2) across the wear durations noted above. In Section 3.3, we then examine the correlations between the gait and sway measures and PRMs of balance confidence and fatigue for wear durations ranging from 1 day to 2 weeks. Finally, in Section 3.4, we present findings from the regression analysis.

The results of the Diff and ICC stages of the wear duration analysis for the gait and sway measures are summarized in Table 1 for a baseline wear duration of 1 week. This table reports a percentage of features that have strong ICCs, in the ICC column, and percentage of features that do not have significant differences in the Diff column. Percentages were used to report the results in a simple frame of reference.

### 3.1. Difference Testing and Intra-Class Correlation for Gait Measures

The results of Table 1 suggest that an adequate median of stability, asymmetry, and temporal gait measures can be obtained from 1 day of data in this sample, as there were no significant differences between gait measures at those wear durations and all ICCs were strong. However, for the 95th percentile, only 80% of the gait measures have strong ICCs (RMS_AP and stance duration are weak, see Table A3 for detailed results). For CV, we observed that only 60% of gait measures have strong ICC from 1 day (Double Support Duration, Stance, Stride, and Swing Duration are weak) and one significant difference (Swing Duration). Increasing the wear duration to 2 days eliminated these weak ICCs, aside from one weak CV of Swing Duration ICC, and the significant difference for the temporal, stability, and asymmetry gait measures relative to baseline in this sample of PwMS. Notably, as we examined the comparisons of wear durations of 1 week and longer, we saw that some gait measures exhibit reduced ICC strength (significant differences are not detected) for wear durations of 4 and 5 weeks. This reduction in ICC is still observed in bootstrapped samples, suggesting that a reduction in sample size is an unlikely explanation.

Interestingly, we found that at least 2 days of monitoring are needed for entropy ratio measures (Entropy Ratio, and Entropy Ratio Asymmetry) and that 1 full week is needed for Lyapunov exponent measures (Lyapunov Exponent AP, Lyapunov Exponent ML) of gait stability in PwMS (see Table A3). These gait measures likely required longer wear durations since they can only be computed from walking bouts of at least 30 s (entropy) or 60 s (lyapunov exponent), effectively limiting the number of observations of these measures each day. Comparing weekdays to weekends, we observed strong agreement between timeframes; however, we observed that the Lyapunov exponent features and double support duration did not exhibit strong ICCs (see Table A3). With the presented information, we cannot speculate on why these differences were observed.

### 3.2. Difference Testing and Intra-Class Correlation for Postural Sway Measures

For postural sway measures, the results of Table 1 indicate that sway may require longer durations than gait. Only 38% of measures show strong ICCs for their median (46% for 95th percentile and 8% for CV) and 23% show differences in their CV for 1 day of data. Two days of data improved the results (no significant differences, median ICCs all strong, 92% of 95th percentile, and CV ICCs are strong), and all measures had strong ICCs and no significant differences after 3 days. The features that fail to show a strong ICC for 2 days of data are 95th percentile of Sway Area and CV of the Mean Period. Moving to longer comparisons, including 3 days, and 1 week to longer periods, we found that the data remain consistent with no significant differences, except for some features demonstrating weak ICCs for CV comparing 1-week to longer durations. Unlike the gait measures, we did not observe any difference between weekdays and weekends. We speculate that postural sway measures require more data than gait measures since the sway features have a wider distribution of values and/or we only consider standing bouts that were 30 s or longer, thereby reducing our observations of these features. The bootstrapped power analysis suggests that the observed ICCs would slightly increase (<10%) with a larger sample size; however, this small increase would not change our conclusions regarding the number of days required.

### 3.3. Correlation of Gait and Sway Features to PRMs

**Figure 2:**
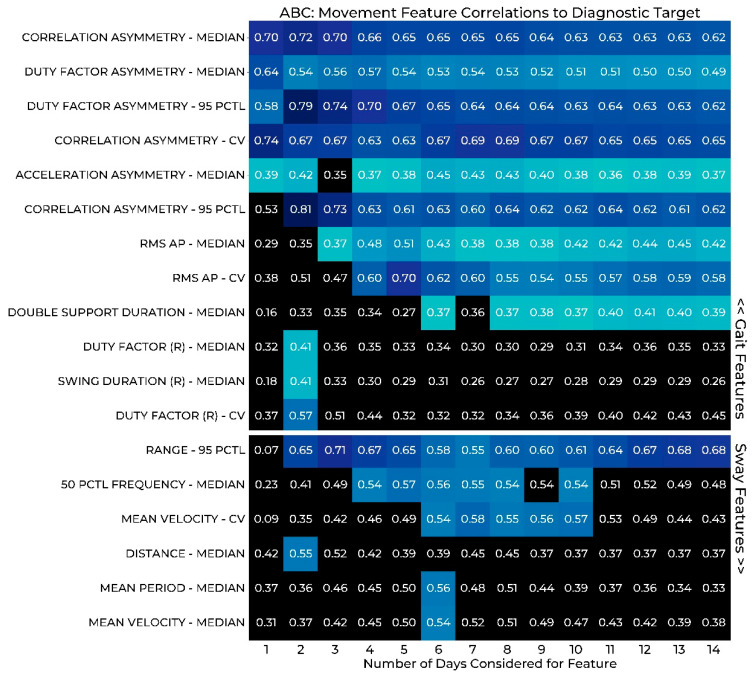
Spearman correlation between Activity-Specific Balance Confidence (ABC) and measures of gait and postural sway by number of sensor-wear days. Cells shaded in blue represent significant correlations (more green = weaker, more blue = stronger) and cells shaded in black were not significant. PCTL: Percentile; CV: Coefficient of Variation; RMS: Root Mean Square; AP: Anterior-Posterior; ML: Medial-Lateral.

**Figure 3:**
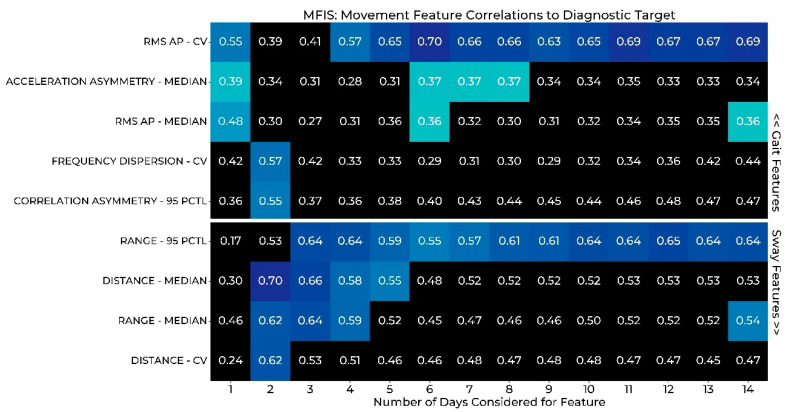
Spearman correlation between Modified Fatigue Impact Scale (MFIS) and measures of gait and postural sway by number of sensor-wear days. Cells shaded in blue represent significant correlations (more green = weaker, more blue = stronger) and cells shaded in black were not significant. PCTL: Percentile; CV: Coefficient of variation; RMS: Root mean square; AP: Anterior-posterior; ML: Medial-lateral.

The results of Stage 3 of the wear duration analysis are reported in the heat maps of Figure 2 and Figure 3, where gait and postural sway measures are correlated with PRMs of balance confidence (Figure 2) and fatigue (Figure 3). Significant correlations are indicated with colored boxes, while those in black do not reach significance. Only features that display significance for at least one wear period are depicted.

Considering the correlations between the measures of gait and postural sway and balance confidence (Figure 2), we observed that the amount of time required to establish a steady significant correlation varies by feature. Both the median and 95th percentile of gait asymmetry measures provided reliable correlations with only 1 day of data and the strongest correlations were observed within the first 2 days of data for most. In the sway features, we observed that 2 days were required to establish a significant correlation with Range, and 4 days were required to establish significant correlations with the 50th percentile frequency. The fluctuation of significance observed in other features may suggest that these features were not as consistent across a longer period. Interestingly, the most significant correlations with the ABC survey occurred with the median or 95th percentile of the features, not the CV. This may suggest that the ABC survey is not sensitive to variation in these gait and sway features and instead is related to more extreme values and typical values of participants during walking and standing activities.

In contrast to the PRM of balance confidence, for the correlations between the measures of gait and postural sway and PRM of fatigue (Figure 3), we did not find a steady nor significant relationship between MFIS and gait asymmetry. As with the ABC, range is reliably established with only 2 days of data. Two new consistent relationships that emerged from this analysis are sway distance and the gait feature RMS AP, which provide a reliable correlation after at least 2 and 4 days of data, respectively.

Considering the correlation results to both PRMs, unsurprisingly we find different results for each survey. This idea makes sense since we would not expect all features to have the same relationship between balance confidence and fatigue or other clinical comparisons. Taking these differences into account, we considered a feature reliable when a significant relationship appears with the survey (ABC or MFIS) and the strength remains similar in the following days. With these criteria, we would consider the gait asymmetry features to be clinically valid with 1 day of data based on the ABC PRM results, although the MFIS relationships were less clear. An example of a relationship we would not consider valid is the correlation between the 95th percentile of correlation asymmetry and MFIS, which is significant at 2 days and then drops out of significance with an approximate 0.18 drop in the correlation coefficient as well, as shown in Figure 3.

### 3.4. Analysis of Factors Impacting Wear Duration

Results from the proposed three-stage wear duration analysis suggest that wear duration is dependent on both the physiological measure being considered (analytical validation aspects) and the underlying disease state (clinical validation) that was being assessed. Specifically, in considering the results of Table 1, there seemed to be support for our hypothesis that wear duration is impacted by how often one observes a given measure during a day and the inherent variability of the measure. For example, the Lyapunov exponent-based measure of gait stability has very few observations per day relative to more traditional gait measures and takes almost a week of data rather than only 2 days. Similarly, postural sway measures inherently have more variability than gait measures and require more days of data. However, to address our hypothesis more directly, we also present the regression results of Table 2. Here, we regress variability (log-transformed CV), number of observations (count), and their interaction on wear duration. All predictors, including an intercept, were at least trend-level significant and the model explains over 40% of the variance in wear duration. Based on the model coefficients, one can see that our hypothesis is supported. Controlling for count, an increase in measure variability will yield a subsequent increase in required wear duration. Similarly, controlling for variability, a decrease in the number of daily observations will yield a subsequent increase in required wear duration. Interestingly, the trend-level interaction term suggests that as variability increases, the relative impact of the number of daily observations on wear duration decreases.

## 4. Discussion

We have proposed a three-stage analysis for determining the wear duration required for capturing impairment and its variability with wearables that align with the current best practices for developing digital medicine technologies that are fit for purpose. The process was used to examine data from a sample of PwMS who have mild to moderate balance and mobility impairment. Herein, we will discuss these results, place them in the context of existing literature, and suggest next steps for future researchers.

Results from the proposed three-stage wear duration analysis are more nuanced than expected and illustrate that each stage provides unique insights. The results of Stages 1 and 2 (difference testing and ICC, Table 1) suggest that 2 days of monitoring are sufficient for most gait and sway features in this sample of PwMS. These results align with prior work in older adults that suggests that 2 to 3 days of data are required for measuring gait speed [22]. However, some measures, such as Lyapunov exponent-based gait stability, can require up to a week of data in this sample. The results of Stage 3 (correlation with PRMs) suggest that 2 to 3 days are needed to find relationships with balance confidence and a week may be needed to find significant relationships with fatigue. Collectively, these results suggest that wear duration is dependent on both the measure being considered and the underlying disease state being addressed. This likely indicates that studies that use only one method to determine the necessary wear duration (e.g., [22,28]) may not be capturing the full picture and could be collecting data for a long period of time, wasting resources and increasing patient burden or not for enough time, yielding unreliable measures or missing key relationships with the underlying disease state being monitored.

Regression results support our hypothesis that wear duration is impacted by how often a given measure is observed during a day and the inherent variability of the measure. These results could be used to inform the deployment of wearables for characterizing digital biomarkers of impairment in several ways. First, given estimates of the number of observations of a measure and its variability, the model could be used to predict an estimate of necessary wear duration. These parameters could be estimated from a small study (2 days of remote wear based on the results presented here) or potentially from values reported in the literature. For example, remote studies of gait often report a number of observed walking bouts each day and variability of the associated gait measures (e.g., see supplementary material of [22]) which could inform this estimate. Moreover, this result could be used to inform protocol changes to reduce the number of required days. For example, if a measure, such as Lyapunov exponent-based gait stability, is critical for a given application, the protocol could include asking participants to engage in a certain number of longer duration walking bouts each day to increase the number of observations.

The proposed approach is in line with developing best practices for ensuring that digital medicine technologies are fit for purpose. As we push to realize the promise of digital medicine enabled by remote patient monitoring with cutting edge wearables, it is critical that the associated measures of impairment are appropriately validated and in the intended patient populations. As demonstrated by the results in Table 1 and Figure 2 and Figure 3, the wear duration analysis presented herein is a key aspect of this validation as the resulting conclusions are impacted by the wear duration considered. Moreover, this multifaceted approach is important as each aspect provides slightly different information and can lead to different conclusions. Importantly, this analysis impacts every application of wearables in digital medicine and can inform the use of these technologies for informing clinical care or as novel endpoints in clinical trials. In the specific studies of gait and postural sway, these results suggest that studies aiming to capture remote assessment of variability likely need only 2–3 days to adequately assess these parameters per monitoring period. This finding may alter the study design to include several 2–3 days of snap shots over a long period of time rather than one long block of monitoring.

There are several limitations to this study, including the limited sample size, and lack of demographic and geographic diversity of the PwMS. We suspect that some of the correlations that alternate between significant and not significant may resolve to a cleaner relationship and firmer conclusions with a larger sample size. Additionally, we do not know how long the 2–3 days of monitoring period is valid for. Particularly in PwMS where we observe symptom fluctuations, studies should be conducted to analyze the change between several 2–3 days of monitoring period that occur throughout the year. Finally, our approach requires the collection of a long baseline monitoring period, which may be prohibitive in some populations. In these circumstances, using the regression approach to determine the necessary wear time may be more appropriate.

## 5. Conclusions

Herein, we present an analysis framework designed to establish a minimum duration of wearable sensor data, which is required to estimate features in the free-living environment. This approach combines previously used methods, difference testing, and intra-class correlation, with an analysis of correlations to PRMs. In the present study, we employ this method to find that the intra-feature variance between 2 days in PwMS compared to 1 week of data is low; however, if the desired outcome of the study is strong correlations with clinical assessments and surveys, a longer monitoring period is likely required for optimal results. Furthermore, regression results reveal that the necessary wear time is significantly related to the number of observations and variability.

## Figures and Tables

**Table 1:** Summary of reliability analysis including difference testing and intra-class correlation.

Comparison	Gait ICC			Gait Diff			Sway ICC			Sway Diff		
	Median	95th P	CV	Median	95th P	CV	Median	95th P	CV	Median	95th P	CV
1 Day vs. 1 Week (n = 22)	100	80	60	100	100	80	38	46	8	100	100	77
2 Days vs. 1 Week (n = 22)	100	100	90	100	100	100	100	92	92	100	100	100
3 Days vs. 1 Week (n = 22)	100	100	100	100	100	100	100	100	100	100	100	100
2 Weeks vs. 1 Week (n = 22)	100	100	100	100	100	100	100	100	85	100	100	100
3 Weeks vs. 1 Week (n = 21)	100	100	100	100	100	100	100	100	100	100	100	100
4 Weeks vs. 1 Week (n = 21)	100	90	80	100	100	100	100	100	100	100	100	100
5 Weeks vs. 1 Week (n = 21)	100	90	90	100	100	100	100	100	85	100	100	100
6 Weeks vs. 1 Week (n = 19)	100	100	100	100	100	100	100	100	85	100	100	100
Weekday vs. Weekend (n = 22)	100	90	90	100	100	100	100	100	100	100	100	100

Summary results of rank sum difference testing (Diff) and intra-class correlation (ICC) for gait (10 parameters) and sway (13 parameters) measures in persons with multiple sclerosis (MS). For Diff, values are percentage of features that did not have a significant difference across wear durations. For ICC, values are percentage of features that had strong (≥0.70) ICC between the compared wear durations. Lyapunov Exponent AP, Lyapunov Exponent ML, Entropy Ratio, and Entropy Ratio Asymmetry are not included in this summary analysis. Data used to inform this table are reported in Appendix A.

**Table 2:** Wear duration time regression analysis.

Coefficient	Estimate	Standard Error	*p*-Value
Intercept	4.41	0.53	**<0.01**
Log CV	0.86	0.41	**0.047**
Count	−0.011	0.0029	**<0.01**
Interaction (Log CV × Count)	−0.0040	0.0020	0.054
R-Squared: 0.46; Adjusted R-Squared: 0.39			
Number of Observations: 27			

Regression analysis of days required for each feature using the log of the coefficient of variation (CV) and feature count computed from 2 days as predictors: Days Required ~1 + Log CV + Count + Interaction. Bolded *p*-values represent significant predictors at a 0.05 significance threshold.

## Data Availability

Not applicable.

## Appendix A

### Appendix A.1. Difference Testing Results

**Table A1:** Gait feature rank sum difference testing in persons with MS.

Gait Feature (14 Total)	1D vs. 1W	2D vs. 1W	3D vs. 1W	WE vs. WD	1W vs. 2W	1W vs. 3W	1W vs. 4W	1W vs. 5W	1W vs. 6W
Acceleration Asymmetry									
Correlation Asymmetry									
Double Support Duration	CV								
Duty Factor									
Duty Factor Asymmetry									
Entropy Ratio									
Entropy Ratio Asymmetry									
Frequency Dispersion ML									
Lyapunov Exponent AP									
Lyapunov Exponent ML									
RMS AP									
Stance Duration									
Stride Duration									
Swing Duration	CV								
Number of Significant Differences	2-CV	0-CV	0-CV	0-CV	0-CV	0-CV	0-CV	0-CV	0-CV
	0-M	0-M	0-M	0-M	0-M	0-M	0-M	0-M	0-M
	0–95th P	0–95th P	0–95th P	0–95th P	0–95th P	0–95th P	0–95th P	0–95th P	0–95th P

Difference testing of gait features for PwMS. A significant difference of the feature at the timeframe is denoted by CV, M or 95th P for Coefficient of Variation, Median, and 95th Percentile, respectively. The timeframes are abbreviated as follows: 1D = 1 Day, 2D = 2 Days, 1W = 1 Week, WE = Weekend, WD = Weekday, etc. If the box is empty, no significant difference was found. The significance threshold was 0.05.

**Table A2:** Sway feature rank sum difference testing in persons with MS.

Sway Feature (13 Total)	1D vs. 1W	2D vs. 1W	3D vs. 1W	WE vs. WD	1W vs. 2W	1W vs. 3W	1W vs. 4W	1W vs. 5W	1W vs. 6W
Area	CV								
Centroidal Frequency									
Distance									
50th Percentile Frequency									
95th Percentile Frequency									
Frequency Dispersion									
Jerk									
Mean Period	CV								
Mean Velocity									
Path									
Power	CV								
Range									
RMS									
Number of Significant Differences	3-CV	0-CV	0-CV	0-CV	0-CV	0-CV	0-CV	0-CV	0-CV
	0-M	0-M	0-M	0-M	0-M	0-M	0-M	0-M	0-M
	0–95th P	0–95th P	0–95th P	0–95th P	0–95th P	0–95th P	0–95th P	0–95th P	0–95th P

Difference testing of sway features for PwMS. Timeframes are abbreviated as follows: 1D = 1 Day, 2D = 2 Days, 1W = 1 Week, WE = Weekend, WD = Weekday, etc. A significant difference of the feature at the timeframe is denoted by CV, M or 95th P for Coefficient of Variation, Median, and 95th Percentile, respectively. If the box is empty, no significant difference was found. The significance threshold was 0.05.

### Appendix A.2. ICC Results

**Table A3:** Intra-class correlation wear duration gait analysis in persons with MS.

Gait Feature (14 Total)	1D vs. 1W	2D vs. 1W	3D vs. 1W	WE vs. WD	1W vs. 2W	1W vs. 3W	1W vs. 4W	1W vs. 5W	1W vs. 6W
Acceleration Asymmetry	0.92	0.95	0.98	0.96	0.99	0.97	0.96	0.95	0.98
	0.89	0.97	0.98	0.94	0.98	0.97	0.94	0.93	0.98
	0.80	0.90	0.91	0.92	0.98	0.90	0.91	0.89	0.94
Correlation Asymmetry	0.96	0.98	0.99	0.98	1.00	0.99	0.98	0.97	0.96
	0.75	0.90	0.95	0.91	0.98	0.96	0.95	0.96	0.95
	0.94	0.96	0.98	0.95	0.98	0.98	0.96	0.93	0.96
Double Support Duration	0.95	0.96	0.98	0.93	0.99	0.98	0.87	0.89	0.96
	0.84	0.83	0.91	***0.38***	0.93	0.88	0.74	***0.48***	0.72
	***0.62***	0.84	0.98	***0.60***	0.96	0.92	0.87	0.78	0.79
Duty Factor	0.96	0.98	0.99	0.98	0.98	0.97	0.91	0.95	0.93
	0.70	0.94	0.97	0.96	0.99	0.96	0.95	0.86	0.89
	0.80	0.91	0.92	0.87	0.97	0.93	0.84	0.76	0.83
Duty Factor Asymmetry	0.96	0.97	0.99	0.98	0.99	0.99	0.97	0.98	0.97
	0.79	0.97	0.96	0.96	0.99	0.99	0.95	0.97	0.96
	0.89	0.94	0.97	0.96	0.97	0.97	0.95	0.94	0.96
Entropy Ratio	-	0.84	0.85	0.71	0.98	0.95	***0.62***	-	-
	-	0.91	0.96	0.91	0.98	0.97	0.81	-	-
	-	0.82	0.91	0.90	0.87	0.90	***0.53***	-	-
Entropy Ratio Asymmetry	-	0.97	0.96	0.88	0.95	0.94	0.92	-	-
	-	0.83	0.85	***0.60***	0.86	0.87	0.78	-	-
	-	***0.45***	0.72	***0.32***	0.91	0.85	***0.39***	-	-
Frequency Dispersion ML	0.96	0.95	0.97	0.94	0.99	0.99	0.93	0.89	0.79
	0.76	0.93	0.95	0.93	0.99	0.99	0.96	0.89	0.96
	0.87	0.94	0.98	0.87	0.98	0.98	0.91	0.84	0.94
Lyapunov Exponent AP	-	-	-	***0.57***	0.99	0.97	0.83	***0.66***	0.89
	-	-	-	0.71	0.97	0.97	0.73	***0.44***	0.93
	-	-	-	***0.02***	***0.00***	***0.00***	***0.08***	***0.19***	***0.00***
Lyapunov Exponent ML	-	-	-	0.79	0.84	0.88	0.83	0.84	0.92
	-	-	-	***0.57***	0.98	0.97	0.80	***0.60***	0.82
	-	-	-	***0.15***	***0.57***	***0.07***	***0.53***	***0.00***	***0.00***
RMS AP	0.83	0.95	0.96	0.94	0.98	0.97	0.88	0.94	0.95
	***0.68***	0.93	0.95	0.87	0.97	0.92	0.86	0.87	0.90
	0.83	0.88	0.94	0.86	0.95	0.95	***0.64***	0.83	0.91
Stance Duration	0.89	0.95	0.98	0.89	0.99	0.98	0.93	0.94	0.98
	***0.53***	0.96	0.96	0.90	0.99	0.98	0.94	0.88	0.92
	***0.57***	0.94	0.96	0.85	0.97	0.96	0.78	0.85	0.88
Stride Duration	0.92	0.95	0.99	0.90	0.99	0.99	0.96	0.98	0.99
	0.84	0.92	0.95	0.88	0.98	0.97	0.95	0.88	0.96
	***0.45***	0.90	0.96	0.81	0.97	0.95	0.84	0.90	0.94
Swing Duration	0.97	0.98	0.99	0.96	0.99	0.98	0.93	0.96	0.97
	0.82	0.91	0.92	0.92	0.96	0.93	0.82	0.80	0.87
	***0.38***	***0.65***	0.78	0.72	0.94	0.86	***0.58***	***0.59***	0.72
Number of Strong Correlations	***10***	12	12	13	14	14	13	11	12
	***8***	12	12	11	14	14	14	9	12
	***6***	10	12	10	12	12	8	9	10

Intra-class correlation of gait features between differing timeframes in PwMS. Non-shaded cells are the correlation between feature medians, grey shaded cells are the correlation between feature 95th percentiles, and blue shaded cells are the correlation between feature coefficient of variation (CV) values. Features and/or timeframes are abbreviated as follows: AP: Anterior-posterior; ML: Medio-lateral; RMS: Root mean square; 1D = 1 Day, 2D = 2 Days, 1W = 1 Week, WE = Weekend, WD = Weekday, etc. Correlations are considered strong if the reported value is greater than or equal to 0.70, values below are bolded and italicized.

**Table A4:** Intra-class correlation wear duration sway analysis in persons with MS.

Sway Feature (13 Total)	1D vs. 1W	2D vs. 1W	3D vs. 1W	WE vs. WD	1W vs. 2W	1W vs. 3W	1W vs. 4W	1W vs. 5W	1W vs. 6W
Area	***0.49***	0.86	0.94	0.93	0.96	0.94	0.84	0.87	0.93
	***0.40***	***0.38***	0.95	0.91	0.98	0.95	0.92	0.81	0.96
	***0.46***	0.81	0.89	0.85	0.92	0.90	0.88	0.86	0.91
Centroidal Frequency	0.84	0.96	0.99	0.95	1.00	0.99	0.97	0.98	0.99
	0.73	0.90	0.97	0.88	0.99	0.99	0.96	0.97	0.98
	***0.49***	0.90	0.95	0.91	0.96	0.96	0.93	0.92	0.95
Distance	0.75	0.94	0.97	0.94	0.97	0.97	0.83	0.89	0.94
	***0.46***	0.86	0.95	0.76	0.96	0.92	0.89	0.88	0.92
	***0.47***	0.94	0.96	0.93	0.96	0.96	0.77	0.86	0.94
50th Percentile Frequency	0.80	0.96	0.99	0.96	0.98	0.97	0.94	0.95	0.96
	0.74	0.94	0.98	0.94	0.98	0.98	0.94	0.96	0.97
	***0.52***	0.84	0.88	0.78	0.96	0.93	0.90	0.83	0.91
95th Percentile Frequency	0.74	0.97	0.99	0.97	0.98	0.96	0.95	0.94	0.96
	***0.64***	0.94	0.98	0.94	0.98	0.98	0.95	0.97	0.98
	***0.32***	0.71	0.90	0.79	0.95	0.91	***0.56***	0.88	0.89
Frequency Dispersion	***0.64***	0.95	0.96	0.95	0.98	0.98	0.91	0.93	0.97
	***0.66***	0.91	0.96	0.89	0.97	0.95	0.93	0.92	0.94
	***0.46***	0.87	0.93	0.93	0.97	0.96	0.86	0.92	0.95
Jerk	0.85	0.88	0.96	0.92	0.98	0.95	0.71	0.84	0.92
	***0.68***	0.87	0.96	0.87	0.96	0.93	0.76	0.83	0.90
	***0.27***	0.79	0.87	0.90	0.93	0.91	0.87	0.83	0.90
Mean Period	***0.01***	0.97	0.97	0.97	0.90	0.90	0.84	0.83	0.94
	***0.43***	0.78	0.96	0.90	0.94	0.96	0.95	0.72	0.96
	***0.56***	***0.67***	0.87	***0.49***	0.84	0.83	***0.40***	***0.41***	0.75
Mean Velocity	***0.56***	0.97	0.96	0.96	0.97	0.96	0.95	0.94	0.96
	0.85	0.80	0.93	0.94	0.95	0.97	0.94	0.86	0.97
	0.72	0.94	0.97	0.90	0.92	0.90	0.76	0.93	0.88
Path	***0.51***	0.97	0.96	0.98	0.97	0.96	0.95	0.93	0.96
	0.84	0.79	0.94	0.94	0.95	0.97	0.94	0.86	0.97
	***0.62***	0.91	0.96	0.94	0.94	0.93	0.83	0.93	0.91
Power	***0.24***	0.96	0.95	0.98	0.96	0.97	0.97	0.92	0.97
	0.82	0.72	0.94	0.94	0.95	0.97	0.92	0.82	0.97
	0.77	0.91	0.96	0.93	0.94	0.92	0.76	0.92	0.88
Range	***0.65***	0.91	0.94	0.94	0.97	0.97	0.82	0.88	0.96
	***0.48***	0.94	0.97	0.95	0.98	0.96	0.88	0.91	0.94
	***0.39***	0.77	0.87	***0.68***	0.91	0.87	0.85	***0.58***	0.83
RMS	***0.49***	0.97	0.96	0.97	0.97	0.97	0.95	0.93	0.97
	0.83	0.79	0.95	0.94	0.95	0.97	0.93	0.86	0.98
	***0.61***	0.92	0.96	0.95	0.95	0.94	0.93	0.93	0.92
Number of Strong Correlations	5	13	13	13	13	13	13	13	13
	6	12	13	13	13	13	13	13	13
	1	12	13	11	13	13	11	11	13

Intra-class correlation of sway features between differing timeframes in PwMS. Non-shaded cells are the correlation between feature medians, grey shaded cells are the correlation between feature 95th percentiles, and blue shaded cells are the correlation between feature coefficient of variation (CV) values. Timeframes are abbreviated as follows: 1D = 1 Day, 2D = 2 Days, 1W = 1 Week, WE = Weekend, WD = Weekday, etc. Correlations are considered strong if the reported value is greater than or equal to 0.70, values below are bolded and italicized.
